# Pulmonary Embolism Associated With Pneumomediastinum and Pneumoperitoneum After Laparoscopic Myomectomy: A Case Report and Literature Review

**DOI:** 10.7759/cureus.109340

**Published:** 2026-05-21

**Authors:** Kyriaki Cholidou, Margarita G Toumanidou, Ioannis Gkiozos, Ioannis Papapanagiotou, Kyriaki Tavernaraki, Theodoros Mariolis-Sapsakos, Stamatis J Karakatsanis, Dimitrios Nikas, Alexandros Manthas, Fotini Sarropoulou, Evangelos Dimakakos

**Affiliations:** 1 First University Department of Respiratory Medicine, Thoracic Diseases General Hospital "Sotiria", Athens, GRC; 2 Department of Physiotherapy, Metropolitan Hospital, Athens, GRC; 3 Department of Oncology, Thoracic Diseases General Hospital "Sotiria", Athens, GRC; 4 Department of Obstetrics and Gynecology, National and Kapodistrian University of Athens, Athens, GRC; 5 Department of Imaging and Interventional Radiology, Thoracic Diseases General Hospital "Sotiria", Athens, GRC; 6 Department of Anatomy, National and Kapodistrian University of Athens, Athens, GRC; 7 Department of Hematology, Thoracic Diseases General Hospital "Sotiria", Athens, GRC; 8 Department of Nursing, National and Kapodistrian University of Athens, Athens, GRC; 9 Third Department of Internal Medicine, Thoracic Diseases General Hospital "Sotiria", Athens, GRC; 10 Department of Anatomy and Physiology, School of Nursing, University of West Attica, Athens, GRC; 11 Center of Prevention, Diagnosis and Treatment of Lymphedema - Lymphatic Diseases for Adults and Children, Metropolitan Hospital, Athens, GRC

**Keywords:** anatomy, laparoscopic myomectomy, pneumomediastinum, pneumoperitoneum, pulmonary embolism

## Abstract

Laparoscopic surgery is a common technique with a minimal risk of consequences for the treatment of gynecological conditions, including uterine leiomyomas. However, in rare instances, serious postoperative complications may occur. We present the case of a 51-year-old patient who underwent a laparoscopic myomectomy without intraoperative complications. The patient experienced acute dyspnea shortly after the surgery, in the absence of other accompanying symptoms. Pneumoperitoneum, pneumomediastinum, and pulmonary embolism (PE) were found during radiological assessment, which included both thoracic and abdominal computed tomography. This case highlights a rare but clinically significant complication that may occur following laparoscopic gynecological surgery, and, to our knowledge, represents the first attempt to suggest a strong association between the coexistence of pneumoperitoneum and pneumomediastinum and PE. Even though venous thromboembolism is still the predominant cause of PE, the potential contribution of perioperative factors warrants further investigation, with the aim of improving the understanding of underlying pathophysiological mechanisms and enhancing patient safety.

## Introduction

Uterine fibroids are the most common benign neoplasms of the female reproductive system and affect a significant proportion of women of reproductive age [1,2]. They are benign tumors that develop in the smooth muscle tissue of the uterus, and their size, shape, and location vary [3].

In recent years, laparoscopy has become the method of choice for the treatment of fibroids. Laparoscopic myomectomy has been established as a minimally invasive, safe, and effective treatment option for symptomatic fibroids, with advantages such as reduced postoperative morbidity, less blood loss, and faster recovery compared to traditional open surgery [4,5]. Despite its minimal invasiveness, laparoscopic surgery carries risks and may be associated with many complications.

The present study describes a case of pulmonary embolism (PE) with simultaneous findings of pneumoperitoneum and pneumomediastinum, following laparoscopic myomectomy. Based on the existing literature, this is the first time that the causal relationship between PE and the presence of air in the mediastinum and abdominal cavity has been attempted.

## Case presentation

The patient provided written and oral informed consent for the publication of this study. A 51-year-old woman presented with episodes of abnormal uterine bleeding. Following clinical and imaging evaluation, uterine fibroids were diagnosed. After assessment of the findings and the patient’s general condition, the treating physicians decided that the most appropriate therapeutic approach was laparoscopic myomectomy. The operative procedure lasted approximately 65 minutes. Carbon dioxide insufflation was initiated at 18-20 mmHg, and intra-abdominal pressure was maintained at 12 mmHg throughout the laparoscopic procedure. Moreover, no anesthetic or surgical complication was recorded during the procedure, and the patient remained hemodynamically stable at all times.

**Figure 1:**
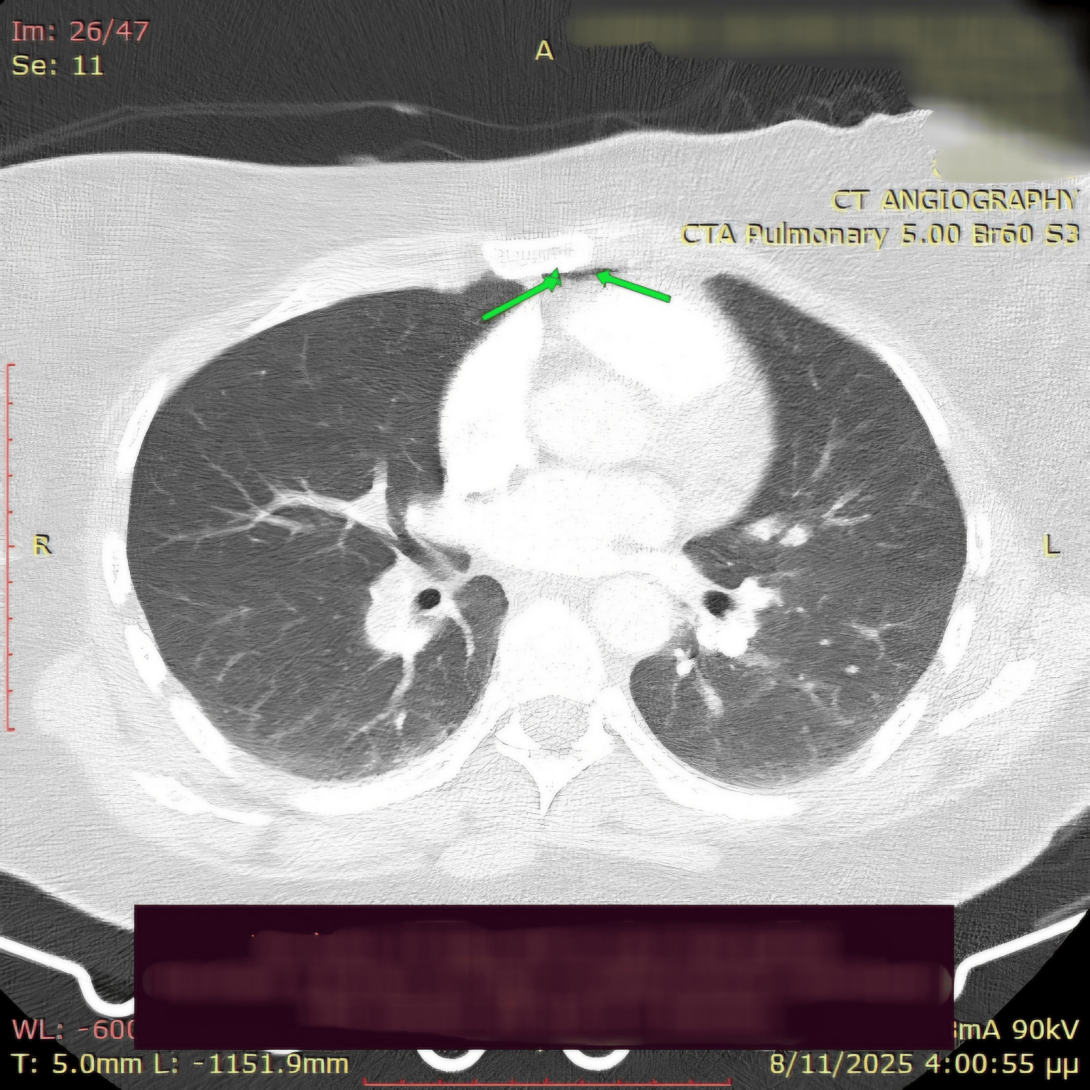
Computed tomography (CT) axial section illustrating free air in the mediastinum consistent with pneumomediastinum (arrows).

**Figure 2:**
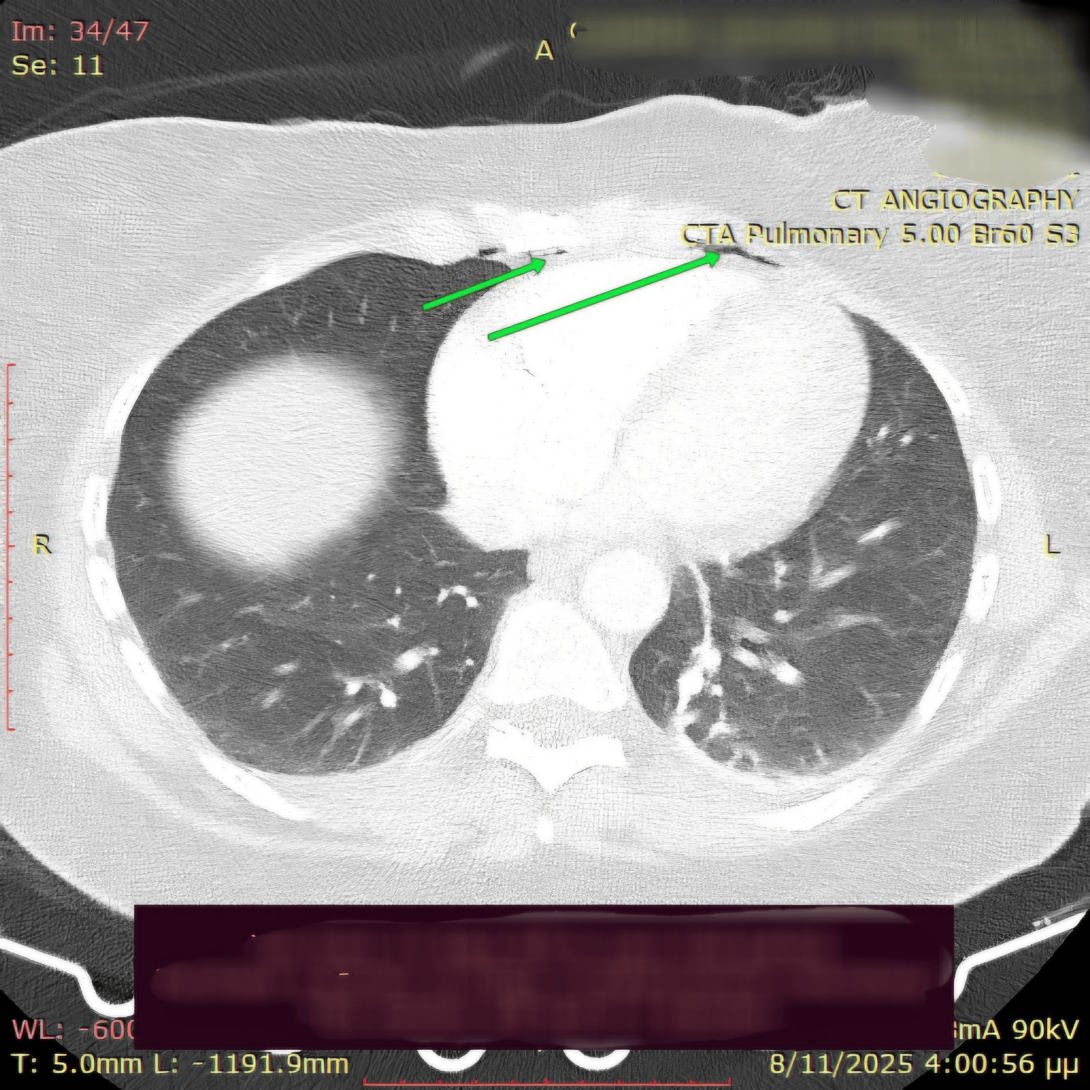
Computed tomography axial image demonstrating pneumomediastinum, with the presence of free air in the mediastinal space (arrows).

**Figure 3:**
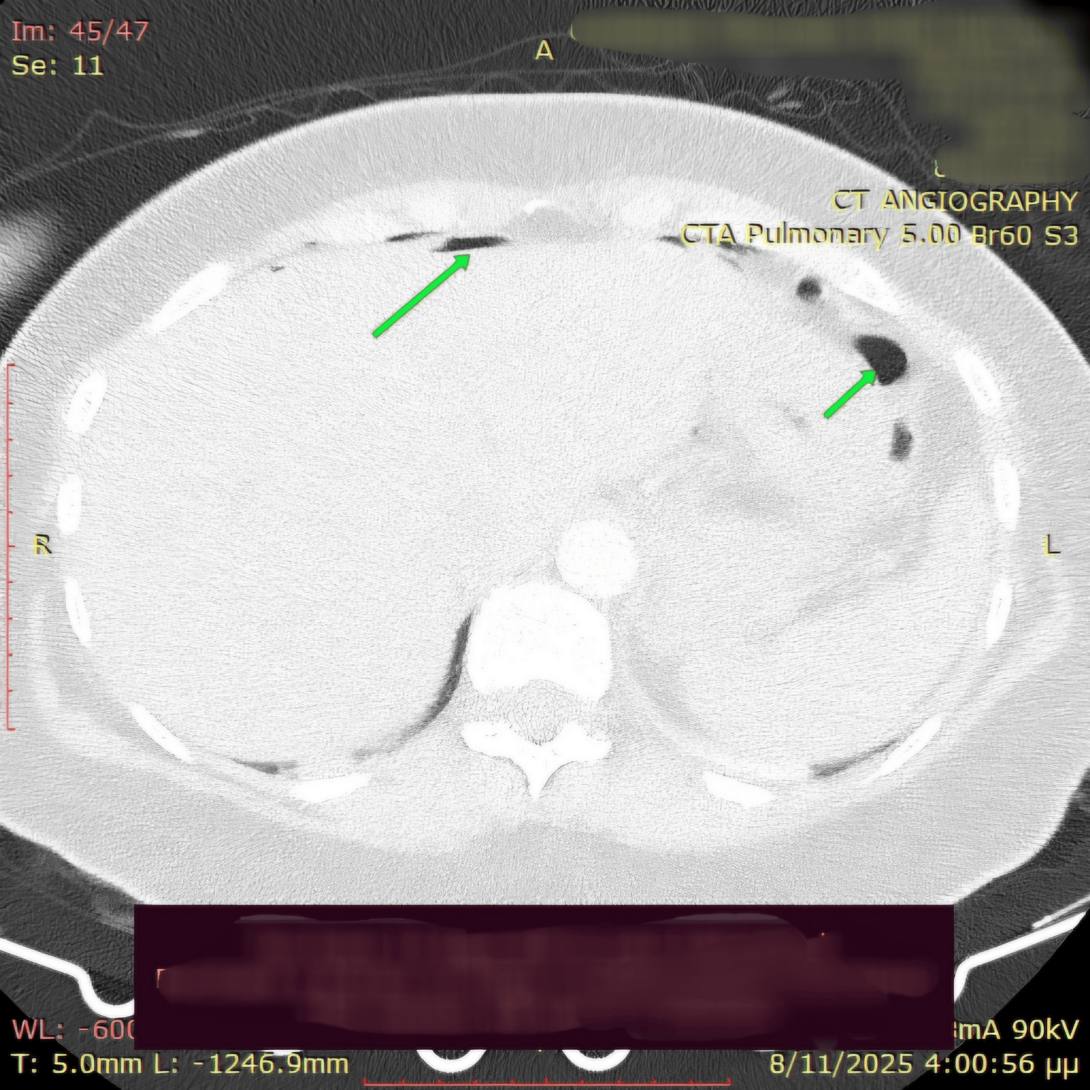
Computed tomography axial image demonstrating pneumoperitoneum, with the presence of free air in the abdominal cavity (arrows).

**Figure 4:**
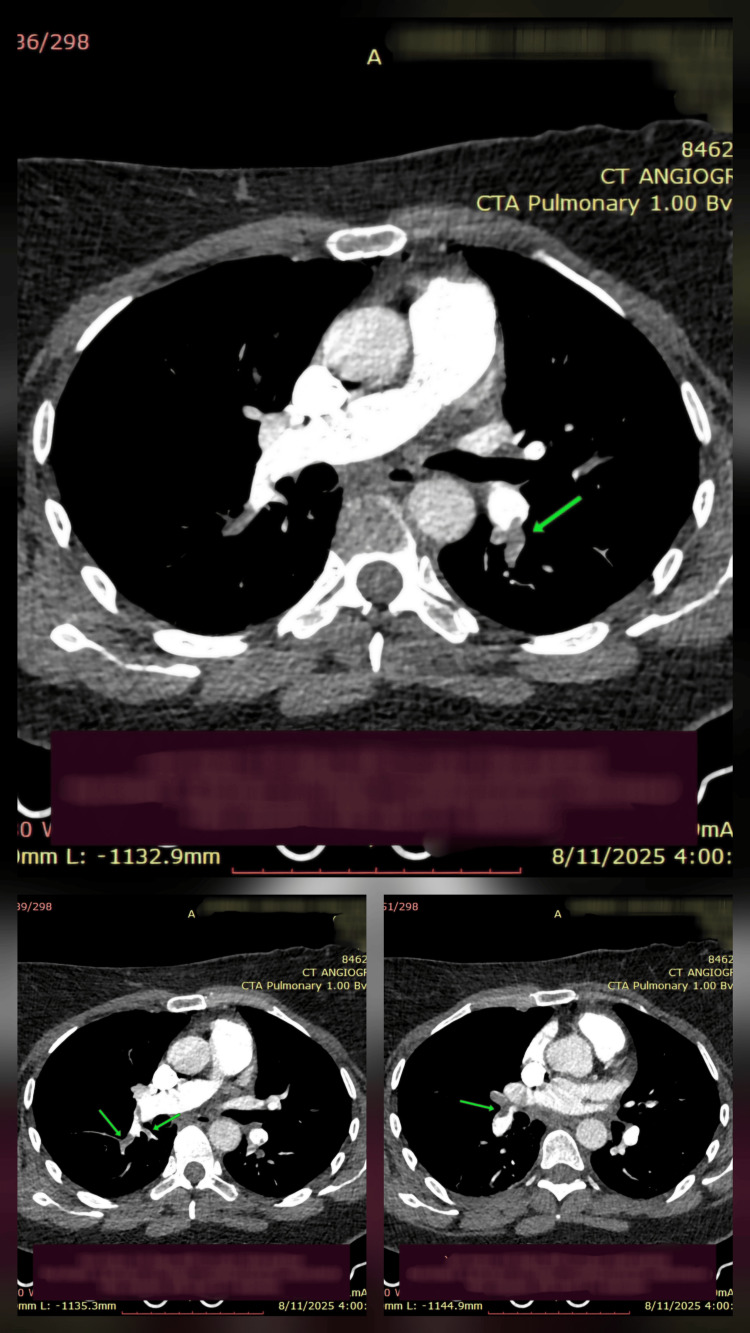
Computed tomography angiography axial image demonstrating pulmonary embolism (arrows).

**Figure 5:**
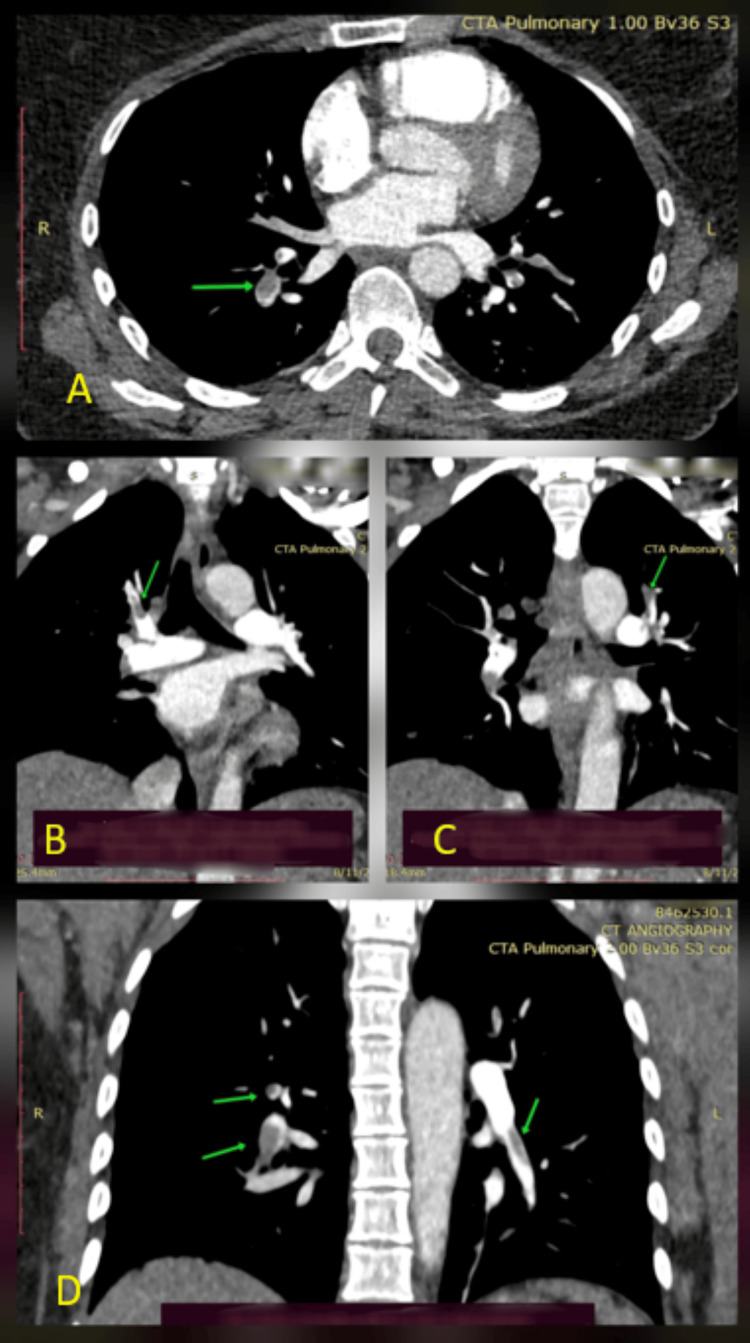
Computed tomography angiography images demonstrating pulmonary embolism (arrows): (A) axial view, (B) coronal view, (C) coronal view, and (D) coronal view.

After two hours of uneventful surgery, the patient developed severe dyspnea, accompanied by a significant drop in oxygen saturation. She underwent computed tomography (CT), which demonstrated PE and the presence of air in the mediastinum and abdominal cavity (Figures 1-5). It is noted that, apart from dyspnea, she did not have any other symptoms such as pain or fever. Furthermore, thoracic and abdominal CT scans and laboratory blood tests did not reveal a ruptured diaphragm or any other disease or pathological problem, which could explain the passage of air from the uterus to the mediastinum.

Only the D-dimer values were found to be elevated, exceeding the normal reference range, as shown in Table 1. It is emphasized that the subsequent thrombophilia and immunological testing, the ultrasound of the heart, and the electrocardiogram were normal. In addition, a duplex ultrasound of the lower extremity veins, as well as an abdominal CT scan, were performed without revealing findings indicative of venous thrombosis. Moreover, her individual history and her family history did not report any disease concerning deep vein thrombosis (DVT), thrombophilia, or any immunological disease.

**Table 1:** Summary of hematological, coagulation profile, thyroid function, and thrombophilia screening results with reference intervals. Ht: hematocrit; WBC: white blood cells; PLT: platelets; PT: prothrombin time; INR: international normalized ratio; APTT: activated partial thromboplastin time; TSH: thyroid-stimulating hormone; Free T4: free thyroxine; ACL IgG: anticardiolipin antibodies immunoglobulin G; ACL IgM: anticardiolipin antibodies immunoglobulin M; anti-β2 GPI IgG: anti-beta-2 glycoprotein I antibodies immunoglobulin G; IgM: immunoglobulin M; FV Leiden: factor V Leiden; MTHFR: methylenetetrahydrofolate reductase

Test	Result	Reference Range
Ht	41%	36-48%
WBC	7789	4.5-11 x 1000/μL
PLT	308.000	150.000-400.000/μL
PT	12 seconds	11.2-15.3 seconds
INR	0.9	0.8-1.2
APTT	29.6 seconds	24-35 seconds
Fibrinogen	264	200-400 mg/dL
D-dimers	6.7	<0.50 mg/L
TSH	2.8	0.4-4.0 mIU/L
Free T4	8.9	4.5-12.5 mg/mL
Homocysteine	11	5-14 mmol/L
lupus anticoagulant	35 seconds	31-42 seconds
ACL IgG	0.4	<10 GPL
ACL IgΜ	0.4	<7 MPL
Anti-β2 GPI IgG	0.5	<0.8 U/mL
Anti-β2 GPI IgM	0.4	<0.8 U/mL
Protein C	93%	70-150%
Protein S	86%	60-140%
Antithrombin (AT-III)	99%	80-120%
FV-Leiden	Negative	-
MTHFR C677T and A1298C	Negative	-
Factor G20210A	Negative	-

**Figure 6:**
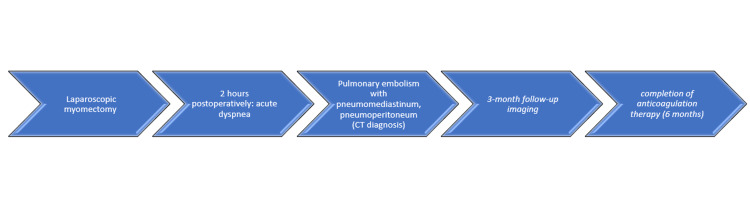
Clinical timeline of the case. The figure was created by the authors using Microsoft PowerPoint (Microsoft Corp., Redmond, WA, USA).

On CT, performed approximately three months after the episode of PE, no findings of PE or abnormal presence of air in the mediastinum and abdominal cavity were identified. Nevertheless, anticoagulant therapy was continued for six months, due to the particular nature of the case and given that the thrombophilia testing was negative. The clinical course of the patient is summarized in Figure 6.

## Discussion

The risk of DVT and PE after laparoscopic procedures is generally considered low, as demonstrated in large studies, where the incidence of PE is approximately 0.2% [6]. However, venous thromboembolic disease is a clinically significant complication and, in addition to general surgery, has been described in women with uterine fibroids. In women with large fibroids, the development of DVT may be due to compression of the pelvic veins, and this compression may cause venous stasis. It may also be attributed to other perioperative risk factors such as immobility and hypercoagulability [1,2]. The hypothesis that large fibroids may compress the pelvic veins is supported by the case of a patient who developed complete occlusion of the common femoral, superficial femoral, popliteal, and great saphenous veins. This event ultimately led to PE in the patient [7].

On the other hand, regarding the perioperative period, it is worth emphasizing that it is a critical time point for the development of thromboembolic events. A typical case is a 51-year-old woman who underwent laparoscopically assisted total hysterectomy and developed acute PE secondary to DVT intraoperatively. This event led to conversion to open surgery [8]. Similarly, in another case of a 53-year-old patient after laparoscopic bilateral salpingo-oophorectomy, a CT scan revealed findings consistent with PE as a result of venous thrombosis. At the same time, pleural effusion appeared, and free air was found in the peritoneal cavity and abdominal wall [9].

Carbon dioxide (CO_2_) leakage is a major complication of laparoscopy, encompassing a wide range of conditions including pneumoperitoneum and pneumomediastinum. The creation of pneumoperitoneum is a necessary step in laparoscopy, but in some cases, CO_2_ can diffuse along peritoneal and retroperitoneal spaces and extend into the thorax and mediastinum [10-12].

The presence of air in the mediastinum after laparoscopic surgery is a rare but documented complication related to the escape and diffusion of CO_2_ through anatomical fascial planes, without necessarily requiring direct injury to the diaphragm [12]. Risk factors for this phenomenon include increased intra-abdominal pressure, prolonged duration of surgery, and high CO_2_ flow rates. In addition, anatomical or congenital weak points in the diaphragm have been suggested as potential pathways for gas passage [13]. Studies have shown that, while subcutaneous emphysema, pneumothorax, and pneumomediastinum are distinct complications, they share a common denominator: CO_2_ leakage during laparoscopy [11].

In gynecological surgery, and especially in laparoscopic myomectomy, pneumomediastinum remains an extremely rare complication. Despite its rarity, it can occur even in the absence of obvious intraoperative trauma. Clinically, it may be asymptomatic or may manifest with chest pain, dyspnea, or cervical subcutaneous emphysema, while in most cases it follows a benign and self-limiting course, with resorption of CO_2_ within 24-48 hours [12,14].

Based on the literature data available to date, there are no published studies describing a causal association between PE and the presence of air in the mediastinum or abdominal cavity after laparoscopic myomectomy as a direct pathophysiological mechanism for causing the thromboembolic complication. Existing reports either report cases of venous thromboembolism as a consequence of known risk factors or isolated descriptions of gas-related complications of laparoscopy, such as pneumoperitoneum, subcutaneous emphysema, and pneumomediastinum. However, these reports have not documented an association with the occurrence of PE.

**Figure 7:**
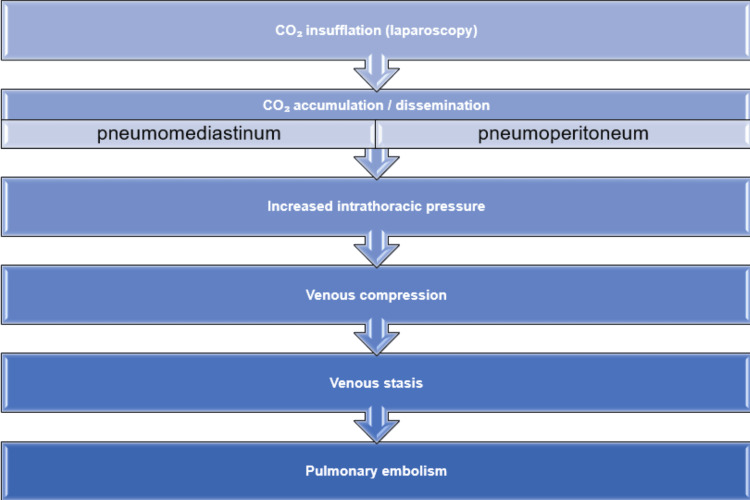
Proposed pathophysiological mechanism. The figure was created by the authors using Microsoft PowerPoint (Microsoft Corp., Redmond, WA, USA).

In this case report, it is hypothesized that the escape and accumulation of CO_2_ in the mediastinum and abdominal cavity may have led to an increase in intrathoracic and mediastinal pressure, with subsequent alteration of the hemodynamics of the great vessels. The increased external pressure on the venous structures could theoretically contribute to venous stasis and impaired venous return, creating conditions that favor the occurrence of PE. Although this mechanism remains hypothetical and has not been documented in the literature, the present observation highlights a strong association between gas-related complications of laparoscopy and thromboembolic events. The proposed pathophysiological mechanism is illustrated in Figure 7.

At the same time, this hypothesis is also strengthened by the fact that the diagnostic testing with duplex ultrasound of the lower limb veins and abdominal CT scan and laboratory blood tests did not reveal findings indicative of venous thrombosis, which makes the classic venous origin of the PE less likely. Thus, the only finding recorded perioperatively was the presence of air in the mediastinum and abdominal cavity.

However, further research is needed to confirm the hypothesis that the occurrence of pneumomediastinum and pneumoperitoneum is associated with PE. This possible association has not yet been documented, but it raises the question of whether changes in intrathoracic and intra-abdominal pressure due to the presence of free air can affect hemodynamics and favor thromboembolic phenomena. Investigation of this mechanism is necessary to better understand the pathophysiology of this complication and to develop effective prevention strategies.

## Conclusions

Although laparoscopic myomectomy is the treatment of choice for uterine fibroids, risks and complications are an integral part of this procedure. This case study gave rise to the hypothesis that the presence of air in the mediastinum and abdominal cavity may be associated with the occurrence of PE due to changes in the pressure of the great vessels. However, further research is required to confirm this hypothesis in order to enable the prevention of complications with respect for patient safety and health.
